# Proximate Compositions, Texture, and Sensory Profiles of Gluten-Free Bario Rice Bread Supplemented with Potato Starch

**DOI:** 10.3390/foods12061172

**Published:** 2023-03-10

**Authors:** Macdalyna Esther Ronie, Hasmadi Mamat, Ahmad Hazim Abdul Aziz, Muhd Khairi Zainol

**Affiliations:** 1 Faculty of Food Science and Nutrition, University Malaysia Sabah, Jalan UMS, Kota Kinabalu 88450, Sabah, Malaysia; 2 Faculty of Fisheries and Food Science, Universiti Malaysia Terengganu, Kuala Nerus 21030, Terengganu, Malaysia

**Keywords:** Bario *Merah Sederhana*, gluten-free, bread, potato starch, texture analysis

## Abstract

Current gluten-free food development trends tend to favour pigmented rice flour. Bario *Merah Sederhana* is a type of red-pigmented rice that is indigenous to Sarawak, Malaysia. This research investigates the nutritional, texture, and sensory properties of gluten-free rice bread produced from a composite of BMS rice flour and potato starch, producing samples referred to as F1 (100:0), F2 (90:10), F3 (80:20), and F4 (70:30). The gluten-free rice bread formulations demonstrated higher ash and crude fibre content and lower carbohydrate content than wheat bread. However, the crude protein content of the bread decreased significantly (*p* < 0.05) with a decreased amount of rice flour, owing to wheat flour containing greater protein. The crumb of rice bread appeared to be darker due to the red pigment of rice flour; in contrast, the crust was lighter than the control sample, possibly due to a lower Maillard reaction. Among rice bread formulations, F4 demonstrated the lowest hardness in dough and bread, as well as the highest stickiness and springiness in dough and bread, respectively. The wheat bread received the highest rating (*p* < 0.05) in the sensory test; nonetheless, among the rice breads, F4 was considered to be an acceptable formulation owing to its high score in colour (7.03), flavour (5.73), texture (6.03), and overall acceptability (6.18). BMS has potential in gluten-free rice breads; the formulation of 70% rice flour combined with 30% potato starch was indicated to be acceptable.

## 1. Introduction

Bread is one of the most widely consumed bakery products worldwide. The market for bakery goods is anticipated to increase from USD 416.36 billion in 2021 to USD 590.54 billion in 2028 [1]. Bread is the most dominant product among bakery goods due to its distinct flavour, texture, and nutritional profile, making it one of the most favoured baked products in the world [2]. The main ingredients used in making bread are wheat flour, water, sugar, shortening, improver, salt, and yeast [3]. Meanwhile, numerous recent studies have focused on incorporating seaweed, amaranth, and fruit juices into wheat bread to improve its nutritional profile, particularly in terms of antioxidants, dietary fibre, and vitamins [4,5,6]. Gluten is the most valuable protein composition in wheat (*Triticum aestivum* L.) flour, which is notable for its viscoelasticity, allowing the bread to create air bubbles and produce a porous structure [7]. However, gluten is a protein that can potentially set off health complications known as gluten-related disorders [8].

In general, gluten-related disorders have been associated with celiac disease (CD), nonceliac gluten sensitivity (NCGS), and an allergy to wheat or grains [9]. Celiac disease is a complex autoimmune disorder induced in genetically susceptible individuals by consuming gluten, causing inflammation in the small intestine. This process is accompanied by the development of particular antibodies and leads to a wide spectrum of gastrointestinal and extra-intestinal symptoms [10]; NCGS is a condition that can be triggered by the consumption of gluten protein, and, lastly, wheat allergy is an immunologic reaction to wheat proteins especially common among children [11]. Currently, a gluten-free diet is the only effective treatment for gluten-related disorders. However, research remains continuous to determine the most appropriate and viable alternative treatment [12]. In addition to those individuals with gluten intolerance, a rising number of consumers prefer to avoid gluten for personal reasons, most notably, the perception that a gluten-free diet is a healthy choice [13,14]. Unfortunately, these are the factors that lead to an increasing demand for gluten-free products [7] and boosted interest in gluten-free products among researchers, consumers, and food manufacturers [15].

In recent years, research into gluten-free bread production has increased significantly [16]. Rice (*Oryza sativa* L.) flour is one of the most commonly utilised flours in commercial gluten-free loaves [16,17,18] due to its accessibility, cost-effectiveness, neutral flavour, and well-known hypoallergenic properties [19,20]. Nevertheless, commercially available gluten-free bread predominantly utilises polished rice; thus, pigmented rice has a limited role in gluten-free bread manufacturing [20]. According to Luo et al. [21], gluten-free bread made with polished rice is likely to be nutritionally deficient due to the removal of the bran layer during processing. On the contrary, gluten-free bread produced from pigmented rice contains better nutritional profiles. This is because the bran layer of pigmented rice contains a greater concentration of dietary fibre, minerals, and bioactive components, improving gluten-free bread’s nutrient deficiencies [22]. In fact, the absence of gluten in gluten-free dough or batter has a major impact on dough rheology as well as end-product quality. Thus, additives such as gums, emulsifiers [23], protein, starches, and flour [20] are commonly incorporated to improve the product’s viscosity.

This study focuses on utilising Bario *Merah Sederhana* (BMS), a red-pigmented rice, as the main ingredient in gluten-free bread. BMS is one of the Bario rice varieties, an indigenous crop in Malaysia. The local ethnic community in the Bario highland of Sarawak cultivates these varieties through traditional techniques and prevents the application of inorganic fertiliser [24,25]. A recent study by Ronie et al. [26] reported that BMS contains 9.43% crude protein, 1.32% crude fat, 0.98% ash, 0.62% crude fibre, and an amylose content of 32.05%. In general, incorporating high-protein rice flour in food reduced the carbohydrate content, thereby lowering the glycaemic load during absorption and digestion [27]. Besides, high amylose-content rice flour was determined to produce decent gluten-free rice bread, due to its good gas-holding ability [28]. In recent years, the consumption of pigmented rice flour has gained more attention due to its beneficial antioxidant properties towards human health [29,30,31], especially in lowering the risk of diseases such as type 2 diabetes, obesity, high blood pressure, cancer, and cardiovascular disease [32,33].

At present, the application of rice flour in gluten-free products is expected. However, the current trend of rice flour use in gluten-free food is more focused on utilising pigmented rice flour rather than nonpigmented rice flour. The reason is that pigmented rice flour has been shown to improve gluten-free food’s nutritional profiles. Hence, this research supports the trend by utilising the red-pigmented rice flour BMS, a locally grown crop from Sarawak, Malaysia, in gluten-free bread. On the other hand, this study encourages the utilisation of locally cultivated crops as ingredients in food products and helps to extend the applications of pigmented rice flour in product development. Therefore, this work aims to provide information on the potential application of BMS supplemented with potato starch in gluten-free bread in the aspect of nutritional profiles, texture properties, and sensory profiles.

## 2. Materials and Methods

### 2.1. Materials

BMS, a primary ingredient, was purchased from Zulkifli Suli Enterprise in Kuching, Sarawak, Malaysia. Castor sugar, salt, low-fat dairy milk, and wheat flour (Cap Sauh) were all acquired from 99 Speedmart in Kota Kinabalu, Sabah, Malaysia. The following ingredients were purchased from Bake With Yen Kolombong, Kian Yap, Lot 126, 127, Lorong Durian 5, Kota Kinabalu, Sabah, Malaysia: shortening, dry yeast, potato (*Solanum tuberosum* L.) starch, xanthan gum, soy (*Glycine max* (L.) Merr.) protein isolate, and bread improver. The rice flour was prepared by drying the rice grains, which were ground to a fine powder for 80 s in a Waring blender (Panasonic MX-898 M, Selangor, Malaysia). The grinding procedure was repeated until finely ground rice flour could pass through a 250-micron sifter (Endecotts Ltd., London, UK). A laboratory sieve shaker (Endecotts Ltd., London, UK) was used to sift the rice flour. The fine rice flour was sealed in airtight bags and kept at 4 °C until further utilisation.

### 2.2. Methods

#### 2.2.1. Dough and Bread Making

The breadmaking procedures were modified slightly from the method described by Kim et al. [34]. The gluten-free rice bread was produced with a 70:30, 80:20, 90:20, and 100:0 ratio of rice flour to potato starch, with the control sample produced entirely of wheat flour. The control sample was made up of wheat flour (100%), sugar (5%), dry yeast (1%), salt (1.5%), shortening (5%), and water (65%). Based on the ratio of rice flour to potato starch, there were four different recipes for gluten-free rice bread. Other ingredients include sugar (6%), dry yeast (1.5%), salt (2%), shortening (4%), water (83%), bread improver (0.019%), xanthan gum (3%), soy protein isolate (1%), and low-fat dairy milk (3%) as shown in Table 1. The rice flour was prepared by cleaning with tap water for five minutes to remove dust and dirt from the kernels. After draining the water, the rice was dried in a drying cabinet (Thermoline Scientific TD-78T-SD, Sydney, Australia) at 40 °C for approximately 12 h. The bread was prepared according to the standard straight dough method by AACC [35] and AACC [36]. To begin, the rice flour was mixed with potato starch for at least 30 s to homogenise it. Then, using a dough mixer with a hook (Kenwood kMIX kMIX50GBL Stand Mixer, Havant, UK), all dry components were combined at low speed for 1 min. During the mixing process, water at 25 °C and low-fat milk were gently added to the flour mixture and mixed for 1 min. The mixer was then switched off, the shortening was added to the dough, and the mixing procedure was continued for another 30 s. The mixing speed was increased to the maximum level. The dough was beaten for 10 min. The dough was placed in baking pans (150 × 50 × 50 mm) and placed in a dough prover at 35 °C for 1 h. Finally, the fermented dough was baked for 25 min; the oven top temperature was set to 165 °C and the bottom temperature was set to 225 °C. After baking, the bread was allowed to cool to room temperature prior to analysis. The breadmaking procedures for the control sample (wheat flour) were all similar, except for the utilisation of the functional ingredients (potato starch, xanthan gum, soy protein isolate, low-fat milk, and bread improver).

#### 2.2.2. Bread Sample Preparation for Analysis

The bread sample was prepared corresponding to the method described by del Carmen Robles-Ramírez et al. [37] with a slight modification. All freshly made bread samples were dried for 12 h in a drying cabinet (Thermoline Scientific TD-78T-SD, Sydney, Australia). The grinding and sifting method of the dried bread samples were carried out as described in Section 2.1. Ground bread sample powder was kept in airtight plastic bags at 4 °C for later analysis.

#### 2.2.3. Proximate Analysis

In general, fresh bread was used as a sample for moisture and ash. Furthermore, the dried bread samples were ground into powder for crude fibre, crude protein, and crude fat analysis. The moisture content of rice flour was determined using the 925.10 AOAC [38] method (the oven drying method). The ash content was analysed using the dry weight basis described in AOAC Method 923.03 [38]. On the other hand, the crude fibre content was determined using the Fibertherm FT12 (Gerhardt, Brackley, UK). The Kjeldahl method, as described in 920.87 AOAC [38], was used to measure the crude protein content of the sample by measuring the protein percentage. This procedure is divided into three stages: digestion, distillation, and titration [39]. Besides, the crude fat content was determined using the fat extraction equipment and the Soxhlet (FOSS SoxhtecTM 2050, Höganäs, Sweden) method described in 920.85 AOAC [38]. The carbohydrate content was estimated by subtracting the moisture, proteins, ash, lipids, and fibre from the dry weight of the samples [40].

#### 2.2.4. Bread Colour Analysis

The colour profiles of the bread samples (crumb and crust) were determined according to the procedure described by Raman et al. [41]. The colorimeter (Hunterlab CalorFlex EZ, Sunset Hills Road, Reston, VA, USA) was used to determine the colour of the bread crusts and crumbs. The bread sample was placed in a glass sample cup until it reached the minimum sample measurement (25 mm); the sample completely covered the bottom of the cup. The crumb and crust region was measured at three different points, and the mean values for each sample were reported. L*, a*, and b* were used to express colour profiles. The L* value represents the level of light (L = 100) or darkness (L = 0), the a* value represents the value of redness (+a) or green hue (−a), and the b* value represents yellowness (+b) or blueness (−b) [42]. The ColorFlex EZ was standardised using the standard white tile (L* = +97.83, a* = −0.43, b* = +1.98) before analysing the sample to avoid errors during colour reading. Each sample’s result was reported as the mean of three replicates.

#### 2.2.5. Texture Profile Analysis (TPA)

Texture profile analysis was performed on both dough and bread products. Parameters analysed on dough samples were hardness, springiness, cohesiveness, and stickiness, whereas bread products were investigated in terms of their hardness, springiness, cohesiveness, and chewiness.

1. Dough

**Figure 1:**
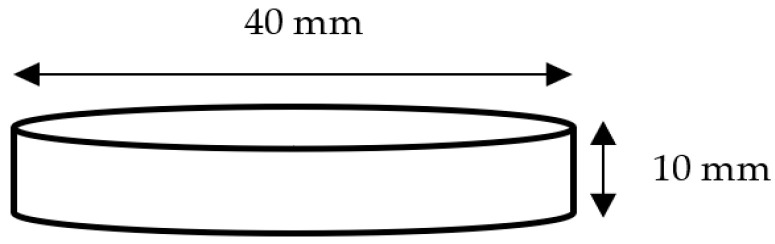
The wheat and rice dough sample measurement for texture profile analysis (TPA).

Texture analysis of dough regarding hardness, stickiness, springiness, and cohesiveness was determined using a texture profile analyser (TA.XT Plus, Texture Technologies, Scarsdale, NY, USA) as described by Mamat and Hill [43], with slight modifications. The sample weighed approximately 10 g and was prepared in round dough disks of 40 mm diameter and 10 mm thickness, using a circular shape-cutter, as shown in Figure 1. A 25 mm diameter cylinder aluminium probe was utilised to compress the dough twice. The test speed was 1.0 mm/s, and the compression distance was 2.5 mm. In addition, the recovery period between 2 strokes was 5 s; hardness, springiness, and cohesiveness values were recorded from the value displayed by the texture analyser software (Texture Exponent 32, Stable Micro System, Surrey, UK). Essentially, dough compression results in the maximum peak force known as hardness. The springiness value was the height for the samples to recover during the time elapsed at the end of the first and second compression [44]. The dough’s cohesiveness was determined by the ratio of the positive force area during the second and first compression [45]. This analysis was run in triplicate for each sample.

On the other hand, stickiness (or adhesiveness) is the work or force necessary to overcome the attraction forces between the product’s surface and the material surface it contacts [46]. The stickiness of dough was performed by referring to the method described by Mamat and Hill [47]. The dough was rounded, sheeted, and moulded into 5 g portions. Next, the dough was inserted into the chamber of stickiness. The compartment was sealed and secured with a screwed-on cap. The initial sample extrusion was scraped away from the surface using a spatula. The top was tightened once again until the dough sample was extruded throughout the chamber at the height of 1 mm. The stickiness of the dough was determined by using an adhesive test with a pre-test speed of 2.0 mm/s, test speed of 2.0 mm/s, and post-test speed of 10.0 mm/s. A 25 mm Perspex cylinder probe was utilised to measure the stickiness of the dough. This analysis was run in triplicate for each sample.

2. Bread

Bread texture analysis was conducted according to the method described by Wu et al. [48], with appropriate modifications. The sample was prepared by cutting the centre of the bread to form a 20 mm (L) × 20 mm (W) × 20 mm (H) piece. The texture properties were examined using a texture profile analyser (TA.XT Plus, Texture Technologies, Scarsdale, USA) equipped with a P/25 aluminium cylindrical probe (25.0 mm in diameter). The pre-test speed was 2 mm/s, the test speed and post-test speed were 1 mm/s, the waiting time was 5 s, the trigger mode was automatic, and the trigger force was 5.0 g.

#### 2.2.6. Sensory Evaluation

A sensory evaluation test was conducted using the nine-point hedonic scale in the sensory laboratory in the Faculty of Food Science and Nutrition (FSMP). In general, the hedonic test is the most straightforward test in which panellists evaluate a few products to determine the best among them [49]. A total of 40 semi-trained panellists comprising students and staff of the Faculty of Food Science and Nutrition (FSMP) participated in this sensory evaluation. Their ages ranged from 21 to 37 years old, and they were non-smokers. First, bread samples were placed in a small sample cup with random three-digit numbers. The bread samples were prepared in 2 cm (L) × 2 cm (W) × 1 cm (H) pieces. Drinking water was provided to the panellists to rinse their mouths before testing each sample. Each member evaluated the bread samples, comprising the control sample (wheat bread) and gluten-free rice breads (F1 to F4), in terms of their colour, flavour, aroma, texture, and overall acceptability. The panel members indicated their scores for each sample in the sensory evaluation form. The nine-level preferences involved in the hedonic test are shown in Table 2.

#### 2.2.7. Statistical Analysis

Statistical Packages for the Social Sciences (SPSS) version 26.0 was used to analyse all experimental data in a completely randomised study design. All experimental values were expressed as mean ± standard deviation. In general, one-way analysis of variance (ANOVA) was used for all studies to determine the statistically significant differences in data between experimental units. In addition, Tukey’s HSD test was used for multiple comparisons. Statistical significance was declared at *p* < 0.05. The sensory evaluation data were analysed by using the Friedman test, and the Wilcoxon signed-rank test was applied as a multiple comparison test to examine whether there was a statistically significant difference between gluten-free bread formulations made with different ratios of rice flour to potato starch. Statistical significance was determined at *p* < 0.05.

## 3. Results and Discussion

### 3.1. Proximate Analysis

The proximate analysis of wheat bread (control sample) and gluten-free rice bread, measuring moisture, ash, crude protein, crude fat, crude fibre, and carbohydrate content, is shown in Table 3.

#### 3.1.1. Moisture Content

The moisture content of all bread ranged from 34.90% to 41.22% (Table 3). Overall, gluten-free rice bread exhibited greater moisture content than wheat bread. This may have been due to the addition of hydrocolloids, which marginally increase the moisture content of freshly baked bread due to the hydrophilic nature of hydrocolloids [50]. In general, the moisture content of gluten-free rice bread formulations statistically showed a significant difference (*p* < 0.05) from wheat bread, except for F3. The moisture content of F3 (36.81%) demonstrated no significant difference (*p* > 0.05) with wheat bread (34.90%). Since F3 contained the lowest moisture content of other rice bread formulations, the possible reason for moisture loss might be due to the result of baking-related evaporation, drying out during the storage period, and the equilibration of moisture between crust and crumb [51]. Previous studies on the gluten-free bread revealed comparable moisture content to our research: 31.36% to 43.66% [52] and 31.5% to 39.1% [53]. According to Ayub et al. [54], the permissible range for the typical moisture content of bread for day-one storage is between 35% and 45%. In the same vein, by referring to the Malaysian Food Regulations 1985 [55], the moisture content in bread is not permitted to exceed 45% at any point throughout the loaf. Therefore, all bread in the current study had acceptable moisture levels.

#### 3.1.2. Crude Ash Content

The ash content ranged from 1.16% to 1.70%. The highest was shown by F1 bread with 100% rice flour. The wheat bread exhibited the lowest ash content, 1.16%; nevertheless, it showed no significant difference (*p* > 0.05) to F4. Overall, gluten-free rice bread possessed greater ash content than control bread. This is due to the rice flour produced from BMS, unpolished rice covered with red-pigmented bran. This was consistent with the findings of prior research, which found that the incorporation of rice bran into bread formulation led to a progressive increase in ash content. [56,57]. Furthermore, a downward trend of ash content starting from F1 to F4 was observed as the percentage of rice flour was reduced. Therefore, it can be concluded that the ash content of gluten-free rice bread depends on the amount of rice flour in bread formulations. Additionally, a comparable result was found in the study of Silva et al. [58], in which the authors reported that the ash content in gluten-free rice bread made from composite red rice flour and cassava flour ranged from 1.11% to 1.34%.

#### 3.1.3. Crude Protein Content

The most significant highest (*p* < 0.05) crude protein content was wheat bread (11.43%). The crude protein content for gluten-free rice bread (F1 to F4) ranged from 8.10% to 10.50%, which statistically showed significant differences (*p* < 0.05) from each other. Starch is the most abundant constituent of wheat flour, followed by proteins. An estimated 80% of protein is composed of gluten-forming protein, a complex mixture of gliadins and glutenins [59]. Saeid et al. [60] reported that the protein content of wheat flour fell between 8.67% and 12.47% and is typically greater than rice flour [61,62]. Thus, this can be the logical reason that wheat bread’s crude protein content was higher than that of gluten-free rice bread. In this research, the crude protein content for F1 (100% RF) was enhanced compared to the raw material, BMS flour (9.43%). To conclude, the increase in crude protein content of the final product may be attributable to the addition of high-protein ingredients, such as soybean protein isolate and low-fat milk. On the other hand, based on Table 3, the crude protein content and amount of rice flour exhibited a directly proportional relationship, whereby the crude protein content increased when the amount of rice flour increased. Hence, this signifies that the amount of rice flour incorporated greatly influenced the crude protein content within gluten-free rice bread. In a study by Chusak et al. [63], wheat bread exhibited a protein content of 10%, whereas pigmented rice bread (Riceberry rice bread) contained a higher protein content compared to white rice bread (Hom Mali rice bread): 8.16% and 7.63%, respectively.

#### 3.1.4. Crude Fat Content

Based on Table 3, the crude fat content of wheat bread (3.72%) and gluten-free rice bread (3.17% to 3.88%) showed no significant difference (*p* > 0.05). According to FoodData Central Search Results [64], conventional white wheat bread contains approximately 3.3% fat; overall, the crude fat content of all bread formulations in this research did not vary much compared to that of the conventional white wheat bread. Mainly, the crude fat content of wheat bread was contributed by the endogenous lipid content of the white flour itself and the addition of shortening in the bread recipe. Similarly, the crude fat content in gluten-free rice bread might synergistically result from incorporating ingredients such as shortening, low-fat milk, and red-pigmented rice flour, which contains a significant amount of crude fat content (1.32%). A comprehensive review analysed by Aguiar et al. [65] reported that the crude fat content of gluten-free rice bread ranges from 1% to 19%. Table 2 reveals that the crude fat content with the lowest value was found in F4. This is likely because F4 is composed of 70% rice flour, probably because F4 contains fewer whole rice grains than other gluten-free formulations, which contributed to the decreased crude fat level. Previous studies reported slightly higher lipid content within gluten-free bread, ranging from 0.20% to 9.57% [63,66]. Variations in breadmaking recipes may account for the observed shifts in fat content.

#### 3.1.5. Crude Fibre Content

The crude fibre content of all formulations ranged from 0.40% to 0.89%. The wheat bread showed the lowest crude fibre content and was significantly different (*p* < 0.05) among formulations. On the other hand, the crude fibre content of gluten-free rice bread demonstrated a gradual downward trend starting from F1 (0.89%), F2 (0.76%), F3 (0.70%), and F4 (0.64%). The percentage usage of rice flour in the formulations affected the downward trend. This is due to the external layer of the rice kernels, which can enhance the crude protein, ash, crude fiber, and crude fat content within baked products [67]. Additionally, the incorporation of potato starch influenced the crude fibre content within the gluten-free rice bread. According to a study performed by Larasati [68], the crude fibre content of potato starch is approximately 0.62%, which is comparable to the crude fibre content of BMS rice flour (0.62%); thus, this likely could increase the crude fibre content of the bread. In the same vein, wheat flour possessed lower crude fibre content, mainly due to the refining process of wheat flour [69]. Therefore, our research proved that the gluten-free rice bread produced from red rice flour could improve the crude fibre content within final products. In addition, several past studies revealed that bread supplemented with maize flour has a high crude fibre level, ranging from 4.96% to 5.86% [70,71].

#### 3.1.6. Carbohydrate Content

The carbohydrate content of wheat and gluten-free rice bread ranges from 42.18% to 48.38%. Wheat bread shows the highest carbohydrate content, with 48.38%, significantly different (*p* < 0.05) from gluten-free rice bread formulations. On the other hand, the lowest carbohydrate content was observed in F1 (42.18%); this formulation utilises 100% rice flour in the bread. Higher carbohydrate content in wheat bread is due to the lower value in other proximate parameters such as moisture, ash, crude protein, crude fat, and crude fibre. For instance, as shown in Table 3, the crude fibre content of wheat bread (0.40%) was significantly (*p* < 0.05) lower than that of gluten-free bread (0.64% to 0.89%). For this reason, the carbohydrate content of wheat bread was the highest among all samples. On the contrary, F1 exhibited the lowest carbohydrate content, because almost all proximate components of F1 were greater than those of other formulations. In addition, the results in Table 3 indicated that 100% rice bread flour contained lower total carbohydrate content compared to gluten-free rice bread produced from the composite mixed flour of rice flour and potato starch. In conclusion, gluten-free bread products made from flour and starches tended to contain high total carbohydrates. These findings corroborate the previous study by Matos and Rosell [72], in which the authors analysed the chemical composition of various gluten-free bread products.

### 3.2. Bread Colour Analysis

**Figure 2:**
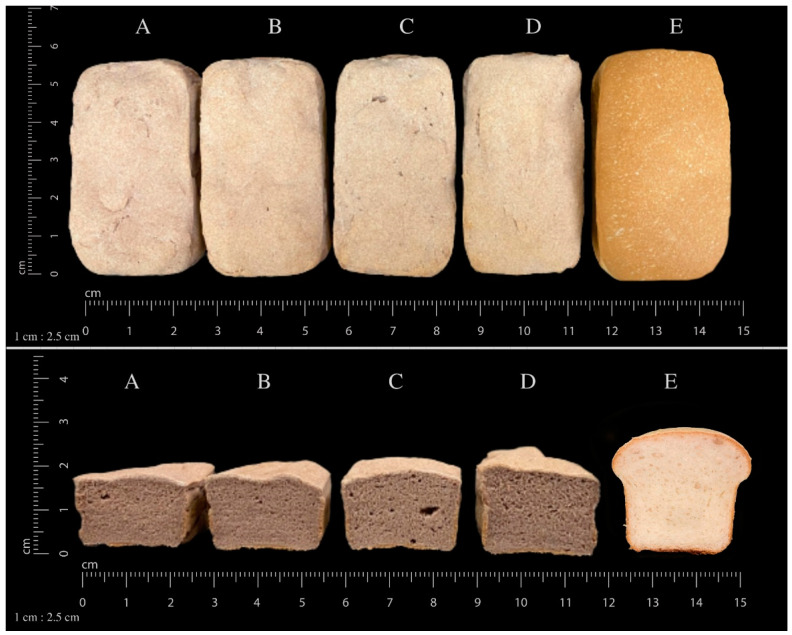
Gluten-free rice bread in comparison to 100% wheat bread (**E**); (**A**): F1 (100% rice flour), (**B**): F2 (90% rice flour + 10% potato starch), (**C**): F3 (80% rice flour + 20% potato starch), (**D**): (70% rice flour + 30% potato starch).

Crumb and crust colour profiles in L*, a*, and b* for wheat and gluten-free rice bread are shown in Table 4. The final products of gluten-free bread are shown in Figure 2.

#### 3.2.1. Crumb

The L* value of the wheat and gluten-free rice crumb indicated a statistical difference (*p* < 0.05). The lightness of the bread crumb was more pronounced in wheat bread because it is colourless compared to the BMS, which includes bran that produces darker flour and crumbs within the final product. Another factor that could shift the colour of red rice products is a process known as “colour-deepening”, which involves the creation of intramolecular bonds in proanthocyanidin as a result of oxidation. In most cases, the heating process causes monomeric anthocyanins to break down into chalcones, which then can be degraded further to a brown colour throughout the heating process [73]. Meanwhile, there was no significant difference (*p* > 0.05) between the L* value of gluten-free rice bread, indicating that the fluctuation amount of BMS did not substantially affect the bread’s lightness. However, a modest increase in F3 and F4′s lightness was still observed. This may have occurred due to the reduction in BMS rice flour while increasing the percentage of potato starch, causing an increase in the bread crumb’s lightness (L* value).

On the other hand, the a* value of the bread’s crumb was more prominent in gluten-free rice formulations. Statistically, wheat and gluten-free rice bread formulation exhibited a significant difference (*p* < 0.05). Overall, gluten-free rice bread showed a high a* value due to the application of rice flour produced from the red-pigmented rice kernels BMS. Based on Table 4, F1 exhibited the highest a* value, with 9.46, due to the usage of 100% rice flour in the formulation. In addition, a descending trend was observed in the a* value as the percentage of rice flour reduced, especially in the F4, which was significantly different (*p* < 0.05) from other gluten-free formulations. The reason is that F4 only contains 70% rice flour, which somehow reduces the bread’s redness value (a*).

For the b* value, the crumb of wheat bread showed a significant difference (*p* < 0.05), which was more yellow compared to the gluten-free bread formulation. This was because wheat grain contains a variety of carotenoids, such as lutein, carotene, zeaxanthin, antheraxanthin, triticoxanthin, and flavoxanthin [74]. Lutein is the most prevalent carotenoid, followed by zeaxanthin, antheraxanthin, and carotene [75]. On the other hand, even though the gluten-free rice bread showed no significant differences (*p* > 0.05) from each other, there was a downtrend in the b* value with a decrease in the amount of rice flour. This is due to the reduction of the yellow hue contributed by the red rice flour.

#### 3.2.2. Crust

There was a statistical difference (*p* < 0.05) between the L* value of wheat and gluten-free rice bread. In contrast to the crumb, the lightness of the bread crust was more prominent in gluten-free rice bread. Several past studies have discovered similar outcomes [58,76,77]. Esteller and Lannes [78] clarified that the light colour of the crust obtained for baked products may have been due to the lower sugar level or higher starch content in the recipe. Moreover, Sciarini et al. [79] speculated that the increased lightness of the hue of the bread crust was due to the addition of hydrocolloid, which can affect the water distribution and, subsequently, influence the caramelisation process and the Maillard reaction. Previous studies by Mandala et al. [80] and Shittu et al. [81] also demonstrated similar results: the addition of hydrocolloids produced bread with a lighter crust. In our research, xanthan gum was the hydrocolloid included in the formulation; xanthan gum is very likely to increase the L* value of the gluten-free rice bread.

The a* value of the bread crust was more noticeable in the wheat bread. All gluten-free rice breads showed no significant difference (*p* > 0.05) from each other. In general, the a* value of the bread crust demonstrated a proportional relationship with the percentage of rice flour. This might be due to the red hue being influenced by the pigmentation of the red rice flour; hence, a reduced amount of rice flour would diminish the a* value of the crust. On the other hand, the redness (a*) of the crust of the wheat bread was higher than that of the gluten-free rice bread, even though it was produced from red rice flour. Gülcan et al. [82] explained that an increase in the redness (a* value) was associated with the Maillard reaction, particularly if the value of L* and a* were in contrast, indicating a high Maillard reaction rate. In our research, the L* value of wheat bread was lower compared to gluten-free rice bread, whereas the a* value was higher in wheat bread than in gluten-free rice bread, denoting the red hue’s prominence in a wheat bread crust. Therefore, it could be concluded that the Maillard reaction was more pronounced in the crust of the wheat bread.

The b* value of the wheat bread crust was significantly higher (*p* < 0.05) compared to the gluten-free bread formulation. This indicates that the crust of wheat bread possessed a greater yellow hue. The high b* value of wheat bread crust indicated a yellower crust, which also resulted from the Maillard browning reaction of reducing sugar and protein in wheat flour [83]. Alternatively, a decrease in the b* value of the gluten-free rice bread crust was observed as the percentage of rice flour was reduced. This may have occurred because decreasing the rice flour percentage might decrease the sugar or protein content in the bread, leading to a lower rate of Maillard reaction, thus producing bread with a lower yellow hue.

### 3.3. Texture Profile Analysis for Dough and Bread

The gluten-free rice dough was subjected to TPA to extract four critical parameters: hardness (g), springiness, cohesiveness, and stickiness. The gluten-free rice bread was analysed for its hardness (g), springiness, cohesiveness, and chewiness. The result is presented in Table 5.

#### 3.3.1. Hardness

Hardness is measured by the amount of force needed to change the shape of a material. Based on Table 5, F1 had the greatest hardness, while wheat dough (control) exhibited the least. There were statistically significant differences (*p* < 0.05) between each sample. Similarly, the highest bread hardness value was indicated by F1 (2846.52 g) and the lowest was by the control (849.13 g). Generally, there was an increase in hardness corresponding to a decrease in rice flour and increase in potato starch. Alam et al. [84] reported that bran may have acted as a reinforcing component, which may have contributed to the increased firmness of the dough. In our study, BMS is a pigmented rice flour encapsulated by bran; thus, bread formulations with higher percentages of rice flour had higher hardness values for both the dough and the bread. On the other hand, another factor that could influence the hardness of the final product is the addition of starch into the formulation. The application of starch in gluten-free bakery formulations has demonstrated a proven capacity to reduce hardness [34,85]. Potato starch can expand better [86], subsequently improving the leavening power of rice dough, leading to greater air sac formation and, finally, softer bread [34]. As can be seen in Figure 2, the formation of air sacs within the F4 crumb was the most obvious among all.

#### 3.3.2. Springiness

The springiness value of bread is directly related to its elasticity [87]. Based on Table 5, the greatest dough springiness was shown by the wheat (0.97) and the lowest was shown by F1 and F2 (0.94). The dough springiness value increased in ascending order from F1 to F4. Although there was no significant difference (*p* > 0.05) between the springiness value of gluten-free rice bread, there was a notable increase in springiness with a decrease in rice flour. Likewise, the bread springiness value was significantly greater (*p* < 0.05) in wheat bread, 1.01. Table 5 shows that the highest gluten-free bread springiness was exhibited by F4. In general, the hydration of gluten-free cereal, such as rice flour does not possess strong foaming properties like wheat flour [88], resulting in fewer air pockets formed during the fermentation process, leading to less springy bread.

#### 3.3.3. Cohesiveness

Wheat dough is significantly (*p* < 0.05) cohesive compared to gluten-free dough due to the presence of gluten protein, which promotes the cohesiveness and elasticity of wheat dough [89]. A directly proportional relationship between the amount of potato starch and the cohesiveness of gluten-free dough indicates that bread dough formulated with a higher amount of potato starch shows an increment in cohesiveness. This may be due to the fine particles and larger surface area of the potato starch. According to Mironeasa et al. [90], fine-particle flour or starch is capable of absorbing a great amount of water during mixing, thereby producing more cohesive dough. In the properties of bread, the degree of cohesiveness determines the crumb’s integrity and the crumb’s capability to collapse before it breaks [85,91]. Bread with a low cohesiveness value indicates the crumb is more susceptible to rupture [92]. Based on Table 5, the most significant (*p* < 0.05) and highest cohesiveness was shown by wheat bread (0.78), and there were no significant differences (*p* > 0.05) between F1 (0.31), F2(0.35), F3 (0.31), and F4 (0.33); this indicates that gluten-free rice bread was more susceptible to rupture than wheat bread.

#### 3.3.4. Stickiness

The stickiness of the dough was significantly higher (*p* < 0.05) in the wheat dough (40.12) due to the presence of gluten protein that promoted the elasticity and adhesive properties within the dough. The dough made from 100% rice flour exhibited the lowest stickiness value (12.70), while the highest stickiness was shown in F4 (17.45). The results indicated an inversely proportional relationship between the percentage of rice flour and the stickiness of the dough. Gluten-free rice dough made from 100% rice flour showed the lowest stickiness value. It is highly likely that the stickiness value was influenced by the usage of potato starch. In general, potato starch has a higher water absorption capacity due to its great swelling power [93,94]. Thus, increasing the percentage of potato starch in the formulation, unfortunately, enhances the water absorption capacity, thereby increasing the stickiness of the dough. In the same vein, previous studies by Tao et al. [95] and Wang et al. [94] determined that potato starch increased the stickiness of noodles and dough, respectively. Both studies concluded that stickiness was affected by the high water absorption capacity of potato starch. Overall, the stickiness of wheat dough is dependent on the gluten network that forms during hydration, whereas the stickiness of gluten-free rice dough is determined by the potato starch’s water absorption ability.

#### 3.3.5. Chewiness

Chewiness is measured as the time needed to chew a sample at a consistent rate of force before it acquires the optimum consistency for swallowing [84]. The chewiness of food is a very essential parameter for different ages of consumers. Chewy foods tend to remain longer within the mouth without instantly breaking apart or dissolving [96]. Based on Table 5, the control sample exhibited the highest chewiness value and the most significant (*p* < 0.05) value, of 669.10, among all samples. On the other hand, no significant differences (*p* > 0.05) were shown among the gluten-free rice bread samples. The chewiness value generally indicated a positive correlation to the percentage of rice flour, which the chewiness of the bread enhances with the increment of the amount of rice flour. On the contrary, the chewiness value of gluten-free rice bread decreased with an increased amount of potato starch. Several previous studies found that the addition of starch to the dough can reduce bread’s chewiness. [34,97]. On the other hand, Islam et al. [70] reported that the introduction of whole grain brown rice flour in bread usually produces baked products with a chewy texture. Therefore, since F1 gluten-free bread was made from 100% rice flour, its chewiness was more pronounced compared to other formulations.

### 3.4. Sensory Evaluation

A hedonic test was carried out to determine the acceptability of each sample. This study implemented a nine-level hedonic test scale to obtain the best formulation for baking gluten-free bread made from BMS rice flour. All formulations were assessed based on colour, flavour, aroma, texture, and overall acceptability. A total of 40 semi-trained panellists comprising undergraduate students (second year and above), postgraduate students, and staff, generally aged between 21 and 37 years old, were involved in this test. The outcome of the sensory evaluation is recorded in Table 6.

Based on the scores presented in Table 6, the control bread received the highest score (7.60), a result between “like moderately” and “like very much”. F4 obtained the second-highest score (7.03), a result of “like moderately”, and the lowest score was acquired by F2. In general, F1, F2, and F3 were less preferred due to a higher concentration of BMS rice flour. The colour of BMS rice flour is brownish, and an increase in BMS rice flour concentration led to a darker bread appearance. As a comparison, F4 exhibited lighter colour due to the lowest content of rice flour (70%). To explain further, a low score for F1, F2, and F3 may be attributable to the fact that the majority of panellists had a strong preference for wheat bread, which shifted the colour preference of bread towards a lighter colour.

Regarding its flavour, wheat bread received the highest score (6.88), a result between “like slightly” and “like moderately”. F4 was the second-highest (5.73) for flavour, after the control, and significantly differed, with a *p*-value more than 0.05 (*p* > 0.05) against the control. The score of F4, 5.73, is associated with “neither like nor dislike” and “like slightly”. Nonetheless, F1, F2, and F3 generally scored lower, ranging between 5.20 and 5.38, conveying a meaning of “neither like nor dislike”. Based on the result obtained, gluten-free breads containing 80% rice flour or more were less preferred by the panellists. The reason is that higher rice flour concentrations might sharpen the final product’s flavour. Filipini et al. [98] revealed that rice cakes made from brown rice flour are more pronounced in flavour, described as “brown rice flavour”, than those made from white rice. Therefore, it can be concluded that an increase in the percentage of brown rice flour consequently intensified the brown rice flavour in the gluten-free rice bread, which was not preferable to the panellists.

The highest rate was acquired by wheat bread with a score of 6.68, corresponding to “like slightly” and “like moderately”. Interestingly, F2 was rated as the second-highest formulation, with a score of 6.08, conveying “like slightly”. However, both were significantly different (*p* < 0.05). In addition, the lowest score among all was received by F3 (5.65), equivalent to “neither like nor dislike” and “like slightly”. Gluten-free rice bread, in comparison to wheat bread, has a more pronounced yeast aroma [99]. This may be why gluten-free rice bread received a lower score than wheat bread. Nonetheless, F2 received a high preference among gluten-free formulations, because a high concentration of rice flour (90%) produces an intense rice powder aroma that somehow increases the liking for this formulation.

Based on the results shown in Table 6, the wheat bread sample obtained the most significantly (*p* < 0.05) score (6.80), conveying to a response of “like slightly” and “like moderately”. F4 received the second-highest rating, with 6.03, corresponding to “like slightly”, and the lowest score was obtained by F2, with a score of 4.83. The majority of panellists rated F2 between “dislike slightly” and “neither like nor dislike”. These results align with the outcome of the texture profile analyser (TPA) for bread in Section 3.3. F1 obtained the lowest rating due to the bread’s extreme firmness. According to the result obtained in the TPA, F1 demonstrated the highest hardness value, 2846.52 g, whereas the lowest hardness value among the gluten-free rice breads was F4, 1658.15 g. For this reason, F4 obtained a good score compared to other gluten-free rice bread. To explain further, a possible factor influencing the bread’s hardness was the amount of potato starch added. Overall, it appears that increasing the amount of potato starch in gluten-free rice bread may soften the texture. In 2015, Kim et al. [34] reported a similar finding: the authors discovered that adding starch to gluten-free rice bread appealed to consumer’s preference for softness.

Most panellists are not consumers of gluten-free bread, and only 5% of the panellists had experienced the consumption of gluten-free bread. Hence, it is undeniable that the highest rating was obtained by the control sample (wheat bread). However, F4 received the highest scores for colour, flavour, texture, and overall acceptability among gluten-free rice breads. Therefore, for the panellists, overall, F4 was the most consumer-acceptable formulation of gluten-free rice bread.

## 4. Conclusions

The potential application of Bario rice flour in gluten-free rice bread was demonstrated. BMS flour is generally capable to improve the nutritional profiles of gluten-free rice bread. The increase in the ash content showed that the compositions related to ash constituents, such as vitamins and minerals, were improved by the utilisation of BMS flour. Moreover, BMS flour increased the crude fibre and lowered the carbohydrate content compared to that of wheat bread. Based on the final products, the crumb colour of wheat bread was lighter than gluten-free rice bread; this is due to red rice flour including a bran layer which, unfortunately, darkens the bread’s crumb. In contrast, the crust of gluten-free bread was lighter than wheat bread due to the low Maillard reaction. Among all gluten-free rice formulations, the F4 dough showed the least hardness and highest stickiness and cohesiveness. This was due to the addition of 30% potato starch, which increased the hydration capacity of the rice dough. The lowest hardness and the increased stickiness in the F4 dough produced the least hardness and high springiness in the final F4 bread. This may demonstrate that low dough hardness and increased stickiness can produce bread with low hardness as well as good springiness. Wheat bread exhibited outstanding texture characteristics compared to gluten-free bread. It was expected that the highest rating would be received by wheat bread. F4 was determined to be the most acceptable formulation among gluten-free breads owing to its colour, flavour, texture, and overall acceptability. To summarise, the application of BMS has potential in gluten-free bread; especially the formulation of 70% rice flour mixed with 30% potato starch. However, several improvements can be implemented in future studies, especially to improve the texture of gluten-free rice bread, making it more comparable with wheat bread. In addition, choosing panellists that were not familiar with gluten-free bread may have increased the panellists’ preference for the control sample.

## Figures and Tables

**Table 1:** The formulation utilised in the preparation of bread.

Baker Ratio (%)	Formulation				
	Control (F1)	F2	F3	F4	F5
Wheat flour	100	0	0	0	0
Rice flour	0	100	90	80	70
Potato starch	0	0	10	20	30
Sugar	5	6	6	6	6
Dry yeast	1	1.5	1.5	1.5	1.5
Salt	1.5	2	2	2	2
Shortening	5	4	4	4	4
Water	65	83	83	83	83
Bread improver	0	0.019	0.019	0.019	0.019
Xanthan gum	0	3	3	3	3
Soy protein isolate	0	1	1	1	1
Low-fat dairy milk	0	3	3	3	3

**Table 2:** The nine-level preferences in the hedonic test.

Score	Preference
1	Dislike extremely
2	Dislike very much
3	Dislike moderately
4	Dislike slightly
5	Neither like nor dislike
6	Like slightly
7	Like moderately
8	Like very much
9	Like extremely

**Table 3:** Proximate value of wheat bread and gluten-free rice bread.

Parameters (%)	Wheat Bread (Control)	F1	F2	F3	F4
Moisture	34.90 ± 0.92 ^b^	41.22 ± 0.70 ^a^	39.85 ± 0.91 ^a^	36.81 ± 0.69 ^b^	40.86 ± 0.79 ^a^
Ash	1.16 ± 0.20 ^c^	1.70 ± 0.05 ^a^	1.67 ± 0.13 ^ab^	1.63 ± 0.20 ^ab^	1.28 ± 0.15 ^bc^
Crude protein	11.43 ± 0.08 ^a^	10.50 ± 0.12 ^b^	9.78 ± 0.19 ^c^	9.02 ± 0.06 ^d^	8.10 ± 0.02 ^e^
Crude fat	3.72 ± 0.37 ^a^	3.50 ± 0.45 ^a^	3.61 ± 0.16 ^a^	3.88 ± 0.43 ^a^	3.17 ± 0.04 ^a^
Crude fibre	0.40 ± 0.06 ^c^	0.89 ± 0.14 ^a^	0.76 ± 0.08 ^ab^	0.70 ± 0.08 ^ab^	0.64 ± 0.05 ^b^
Carbohydrate	48.38 ± 1.23 ^a^	42.18 ± 0.69 ^cd^	44.33 ± 0.73 ^c^	47.96 ± 0.89 ^ab^	45.96 ± 0.76 ^bc^

Notes: mean ± standard deviation (*n* = 3). Mean values in the same row with different superscripts are significantly different with *p* < 0.05 by Tukey’s HSD test. RF = rice flour; F = formulation; PS = potato starch; F1 = 100% RF; F2 = 90% RF + 10% PS; F3 = 80% RF + 20% PS; F4 = 70% RF + 30% PS.

**Table 4:** Colour analysis of wheat bread and gluten-free rice bread.

Samples	Crumb			Crust		
	L*	a*	b*	L*	a*	b*
Wheat (Control)	80.40 ± 0.72 ^a^	−0.06 ± 0.37 ^c^	22.97 ± 0.57 ^a^	68.37 ± 2.86 ^b^	7.4 ± 2.51 ^a^	43.66 ± 2.93 ^a^
F1	49.0 ± 6.63 ^b^	9.46 ± 0.08 ^a^	11.69 ± 0.11 ^b^	79.39 ± 1.11 ^a^	3.6 ± 1.75 ^ab^	28.65 ± 2.93 ^b^
F2	47.69 ± 0.41 ^b^	9.21 ± 0.11 ^ab^	11.44 ± 0.09 ^b^	81.48 ± 2.48 ^a^	2.74 ± 1.04 ^b^	17.98 ± 2.32 ^c^
F3	50.76 ± 0.28 ^b^	9.07 ± 0.03 ^ab^	11.43 ± 0.03 ^b^	81.43 ± 2.71 ^a^	0.31 ± 0.34 ^b^	15.86 ± 1.84 ^c^
F4	50.61 ± 0.19 ^b^	8.71 ± 0.38 ^b^	11.24 ± 0.45 ^b^	83.09 ± 0.57 ^a^	−0.07 ± 0.93 ^b^	13.73 ± 1.5 ^c^

Notes: mean ± standard deviation (*n* = 3); mean values in the same column with different superscripts are significantly different with *p* < 0.05 by Tukey’s HSD test; F = formulation; RF = rice flour; PS = potato; F1 = 100% RF; F2 = 90% RF + 10% PS; F3 = 80% RF + 20% PS; F4 = 70% RF + 30% PS.

**Table 5:** TPA results for gluten-free rice dough and bread.

**Parameters**	**Wheat (Control)**	**Dough**			
		**F1**	**F2**	**F3**	**F4**
Hardness (g)	137.371 ± 18.65 ^e^	656.339 ± 39.02 ^a^	546.812 ± 24.29 ^b^	432.027 ± 22.26 ^c^	354.568 ± 23.93 ^d^
Springiness	0.971 ± 0.00 ^a^	0.941 ± 0.17 ^b^	0.951 ± 0.00 ^ab^	0.947 ± 0.00 ^b^	0.943 ± 0.00 ^b^
Cohesiveness	1.013 ± 0.53 ^a^	0.649 ± 0.35 ^c^	0.732 ± 0.84 ^bc^	0.777 ± 0.01 ^bc^	0.792 ± 0.04 ^b^
Stickiness	40.122 ± 0.60 ^a^	12.695 ± 0.23 ^d^	14.946 ± 0.39 ^c^	16.437 ± 0.71 ^b^	17.454 ± 0.55 ^b^
**Parameters**	**Wheat (Control)**	**Bread**			
		**F1**	**F2**	**F3**	**F4**
Hardness (g)	849.134 ± 115.59 ^c^	2846.519 ± 259.95 ^a^	2352.54 ± 218.41 ^ab^	2100.645 ± 494.57 ^ab^	1658.147 ± 311.19 ^bc^
Springiness	1.005 ± 0.04 ^a^	0.406 ± 0.06 ^b^	0.424 ± 0.07 ^b^	0.431 ± 0.05 ^b^	0.473 ± 0.05 ^b^
Cohesiveness	0.782 ± 0.12 ^a^	0.308 ± 0.02 ^b^	0.35 ± 0.03 ^b^	0.313 ± 0.03 ^b^	0.327 ± 0.04 ^b^
Chewiness	669.099 ± 105.58 ^a^	356.847 ± 68.07 ^b^	348.181 ± 66.22 ^b^	282.85 ± 75.66 ^b^	253.142 ± 29.78 ^b^

Notes: mean ± standard deviation (*n* = 3); mean values in the same row with different superscripts are significantly different with *p* < 0.05 by Tukey’s HSD test; F = formulation; RF = rice flour; PS = potato starch; F1 = 100% RF; F2 = 90% RF + 10% PS; F3 = 80% RF + 20% PS; F4 = 70% RF + 30% PS.

**Table 6:** Scores for sensory evaluation of control (wheat bread) and gluten-free bread made from BMS.

	Formulation				
Attribute	Control (Wheat Bread)	F1	F2	F3	F4
Colour	7.60 ± 1.19 ^a^	6.55 ± 1.65 ^c^	6.38 ± 1.63 ^c^	6.60 ± 1.88 ^bc^	7.03 ± 1.30 ^b^
Flavour	6.88 ± 1.52 ^a^	5.33 ± 1.56 ^bc^	5.38 ± 1.43 ^bc^	5.20 ± 1.59 ^c^	5.73 ± 1.38 ^b^
Aroma	6.68 ± 1.51 ^a^	5.76 ± 1.72 ^bc^	6.08 ± 1.61 ^b^	5.65 ± 1.51 ^c^	5.80 ± 1.44 ^bc^
Texture	6.80 ± 1.65 ^a^	4.83 ± 1.73 ^c^	5.25 ± 1.75 ^c^	5.33 ± 1.65 ^c^	6.03 ± 1.58 ^b^
Overall acceptability	7.15 ± 1.28 ^a^	5.28 ± 1.57 ^c^	5.58 ± 1.24 ^bc^	5.55 ± 1.47 ^c^	6.18 ± 1.30 ^b^

Notes: mean ± standard deviation; mean values in the same row with different superscripts are significantly different with *p* < 0.05 by Wilcoxon signed-rank test; F = formulation; RF = rice flour; PS = potato starch; F1 = 100% RF; F2 = 90% RF + 10% PS; F3 = 80% RF + 20% PS; F4 = 70% RF + 30% PS.

## Data Availability

Not applicable.
